# The Support for Smoke Free Policy and How It Is Influenced by Tolerance to Smoking – Experience of a Developing Country

**DOI:** 10.1371/journal.pone.0109429

**Published:** 2014-10-22

**Authors:** Abdul Rashid, Azizah Ab Manan, Noorlia Yahya, Lailanor Ibrahim

**Affiliations:** 1 Department of Public Health Medicine, Penang Medical College, Georgetown, Penang, Malaysia; 2 Penang State Health Department, Georgetown, Penang, Malaysia; The Ohio State University, United States of America

## Abstract

This cross sectional survey was conducted to determine the support in making Penang UNESCO World Heritage Site (GTWHS) smoke free and to determine the influence of tolerance towards smoking on this support. This is the first phase in making Penang, Malaysia a smoke free state. A multistage sampling process was done to select a sample of respondents to represent the population of GTWHS. Attitude towards smoking was assessed using tolerance as a proxy. A total of 3,268 members of the community participated in the survey. A big majority (n = 2969; 90.9%) of the respondents supported the initiative. Support was lowest among the owners and residents/tenants, higher age groups, the Chinese, men, respondents who had poor knowledge of the places gazetted as smoke free, and respondents with poor knowledge of the health effects on smokers and on passive smokers. The odds (both adjusted and unadjusted) of not supporting the initiative was high among those tolerant to smoking in public areas. Tolerance towards smoking was associated with 80.3% risk of non-support in the respondents who were tolerant to smoking and a 57.2% risk in the population. Health promotion and education concerning the harm of tobacco smoke in Malaysia, which has mainly targeted smokers, must change. Health education concerning the risks of second hand smoke must also be given to non-smokers and efforts should be made to denormalize smoking.

## Introduction

Although smoking prevalence has reduced worldwide, both in men and women, the number of smokers has increased significantly and cigarette smoking is still the chief preventable killer in most countries [1]–[3]. In Malaysia, according to the Global Adult Tobacco Survey (GATS) 2011, 31% of adults aged 15 years and older were current tobacco smokers, more men (43.9%) than women (1.0%) and highest in the 25 to 44 age group. On the average an adult Malaysian smoked 14 cigarettes a day and among those who had ever smoked on daily basis only 9.5% had quit [4]. The World Health Organization (WHO) recommends a 30% relative reduction in current tobacco use in persons age 15 years and above [5].

Burning of tobacco produces a complex mixture of more than 7000 compounds which are risks for a wide range of diseases and premature deaths [2]. Cigarette smokers have an average 10 years reduced life span and the mortality among cigarette smokers is two to three times the mortality among persons who had never smoked [6]. At present most tobacco related deaths are reported in the developed countries but due to the change in smoking trends it is expected that more tobacco related deaths will occur in underdeveloped and developing countries [2],[6]. In Malaysia, tobacco related diseases accounted for the main causes of deaths in the hospitals operated by the Ministry of Health. Heart and pulmonary circulation diseases ranked first due to tobacco related illnesses followed by malignant neoplasms and cerebrovascular diseases [7]. Smoking not only effects morbidity and mortality but also quality of life, participation in workplace and increased health care costs [2].

Second hand smoke is equally if not more injurious to health [8]. In the United States (US) almost 2.5 million non-smoking Americans died from heart diseases and lung cancer due to exposure to second hand smoke. The loss of productivity due to exposure to second hand smoke is estimated to be about USD5.6 billion per year [2]. In Malaysia, it is estimated that 39.8% of adults who worked indoors have been exposed to second hand smoke and 38.4% adults exposed to second hand smoke at home, 84.9% in cafes/coffee shops/bistros, 78.7% in bars/nightclubs, 71% in restaurants, 28.2% in government buildings, 13.6% in indoor shopping complexes and 8.7% in health care facilities [7].

There are several interventions to combat smoking and the hazards of tobacco smoke. One is to target individual's smoking related attitudes or social influence by implementing anti-smoking policies including laws which discourage smoking [2],[9],[10]. Creation of smoke free areas is an effective intervention because it is easier to discourage people from smoking rather than encourage people to quit smoking once they have started [11],[12]. These smoke free areas not only help educate people on the dangers of second hand smoke but also help denormalize smoking by making smoking less socially acceptable and less convenient [2],[13],[14]. According to MPOWER which is a technical package on evidence based tobacco control interventions to reduce tobacco use to protect people from second hand smoke, all public places should be completely smoke free or at least 90% of the population should be covered by complete subnational smoke free legislation. Forty three countries comprising 1.1 billion people have achieved this level [11].

Malaysia became a signatory of the WHO Framework Convention on Tobacco Control (WHO FCTC) on the 23^rd^ September 2003 and ratified it in 2005 and became an official party to the convention in December 2005. In 2011, as part of an initiative to comply with article 8 of the WHO FCTC, a proposal to create a smoke free zone in Penang, Malaysia was submitted to the Penang State Government with the objective to protect people from second hand smoke. The Ministry of Health had earlier gazetted 21 areas to be smoke free and as a start the Penang State Government had gazetted 6 recreational areas as smoke free zones. The current proposal was to include areas which had commercial and residential units. It was decided that George Town World Heritage Site (GTWHS) would be the first phase in making Penang a smoke free state. Studies conducted elsewhere had shown high level of support towards smoke free policies but different social and cultural values affect smoking habits and attitudes towards second hand smoke differently [15]. Thus before implementing the smoke free policy, a preliminary survey was conducted in this area to assess the level of support of the public to the proposal. The objective of this paper is to show the level of support towards the smoke free policy and the influence of tolerance to smoking on it.

## Materials and Methods

### Study design and Location

This cross sectional survey was conducted in Penang UNESCO World Heritage Site. The survey was commissioned by the Malaysian Health Promotion Board and the Penang State Government to determine the public and the stakeholders' support towards making Penang a smoke free state. The survey commenced from the middle of 2012 and ended in April 2013. Penang is one of the 14 states in Malaysia with a multi-ethnic population. It is one of the most densely populated states in the country with a population of 1,561,853. George Town, the capital of Penang, was inscribed as a UNESCO World Heritage Site on 7^th^ July 2008 under Cultural Heritage Site category. This site features residential, commercial buildings, tourist sites and public open areas.

### Sample

Multistage sampling process was done to select a sample of respondents to represent the population of GTWHS. Samples were taken from eating outlets, accommodation & hotels, places of worship, private and government offices, residences, retail & businesses, hawkers, education facilities, places of tourist attraction and public open spaces. Stata was used to calculate the sample size. Because each premise had a different population size, the sample from each premise was taken with a 5% margin of error. Within each premise/location, the type of respondent ranged from owner or senior management, employee, clients and the general public. This allowed the investigators to capture a wide range of respondents from the different levels. However because of the dynamic population, the study unit from each premise was taken based on the willingness to participate in the study.

### Tool

Data were collected using face to face interviews by trained interviewers. The interviewers were trained comprehensively on accurate methods of data collection to avoid variations and to ensure uniformity. A uniform protocol covering the questionnaire, inclusion and exclusion criteria for each question was set up to minimize error and bias. Besides the baseline demographic information, the questionnaire was used to gather information on the support for making GTWHS a smoke free zone. Other independent variables related to the support including knowledge, attitudes and practices were also included in the questionnaire. Attitude towards smoking was assessed using tolerance as a proxy [16]. There were 16 items in the tolerance scale, the respondents were asked if they would tolerate smoking in 16 different public locations. Respondents were considered not tolerant to smoking if they answered they would not tolerate smoking in all the public places. The smoking tolerance items were examined using Mokken scaling to assess for fit to a single underlying dimension of tolerance. All items on the tolerance scale were selected by the Mokken scaling procedure, and the final scale had an H coefficient of 0·77, indicating that the items met the criteria for a unidimensional scale. The scale had high consistency level (Cronbach alpha reliability 0.97). The scale used to assess the tolerance towards smoking is in Appendix S1.

### Analysis

Data were tabulated and cross tabulated. SPSS version 18 was used to analyse the relationship between the variables using Chi square test. Odds ratio was used to quantify the odds of not supporting the proposal and regression analysis to estimate support for smoke free zone using tolerance as predictor. Stata was used to measure the attributable risk in the exposed and in the population. A probability value of P<0.05 was considered to be statistically significant.

### Ethics

The research had received the approval of the Joint Penang independent ethics committee (JPEC 01-13-0014). All respondents were asked to give a written informed consent before starting the interview. The anonymity of the respondents was assured.

## Results

A total of 3,268 members of the community participated in the survey. Most of the participants were interviewed in business premises (n = 771, 23.6%) followed by offices (both government and private) (n = 455, 13.9%) and education premises (n = 404, 12.4%). The majority of the participants were clients/customers/patrons/tourists/general public (44.6%) followed by owners (26.0%), employees (19.9%) and residents/tenants (11.4%). The majority were men (58.8%), age group 25 to 44 (37.6%), Chinese (60.3%), married (61.4%), income group of between RM1001 to 3000 (66.0%) and the highest level of education up to secondary school (51.7%). Most had never smoked (79.4%) and were aware of the national laws prohibiting smoking in certain areas (86.6%) and had good knowledge of places which were smoke free (69.5%). Most had good knowledge of the health effects of tobacco smoke on smokers (86.4%) as well as on passive smokers (81.2%). Although the majority were not tolerant to tobacco smoke (67.3%), a substantial proportion of the participants were tolerant (32.7%).

A big majority of the respondents supported (n = 2969; 90.9%) the smoke free initiative. As shown in Table 1, more than 10% of the owners and residents/tenants did not support the smoke free zone initiative (χ2 = 24.76; P<0.001). The support for the initiative decreased as the age group increased (χ2 = 9.99; P = 0.01). Among the races, Chinese had the highest non-support proportion (χ2 = 18.74; P<0.001). The support for the initiative increased with increasing education levels (χ2 = 45.29; P<0.001), similarly the support increased as the income bracket increased (χ2 = 9.71; P = 0.01). Support was highest among those who never smoked followed by past smokers and lowest among current smokers (χ2 = 219.89; P<0.001). The support for the initiative was lower among the men (χ2 = 16.16; P<0.001), respondents who had poor knowledge of places gazetted as smoke free (χ2 = 30.16; P<0.001), poor knowledge of health effects on smokers (χ2 = 122.59; P<0.001) and among passive smokers (χ2 = 83.48; P<0.001).

**Table 1:** Factors associated with non-support to making GTWHS a smoke free zone.

Variable	N	No support	Support	Chi square/P value
**Respondent Type**				24.76/<0.001
Owner	851	92 (10.8)	759 (89.2)	
Employee	584	38 (6.5)	546 (93.5)	
Clients/customers/patrons/tourists/general public	1459	114 (7.8)	1345 (92.2)	
Residents/tenants	374	55 (14.7)	319 (85.3)	
**Age**				9.99/0.01
≤24	688	61 (8.9)	627 (91.1)	
25–44	1229	91 (7.5)	1125 (92.5)	
45–64	1052	110 (10.5)	942 (89.5)	
≥65	299	37 (12.4)	262 (87.6)	
**Gender**				16.61/<0.001
Men	1923	209 (10.9)	1714 (89.1)	
Women	1345	90 (6.7)	1255 (93.3)	
**Marital Status**				0.38/0.95
Married	2008	186 (9.3)	1822 (90.7)	
Divorce	28	3 (10.7)	25 (89.3)	
Widow/widower	17	1 (5.9)	16 (94.1)	
Single	1215	109 (9.0)	1106 (91.0)	
**Race**				18.74/<0.001
Malay	634	43 (6.8)	591 (93.2)	
Chinese	1970	215 (10.9)	1755 (89.1)	
Indian	462	28 (6.1)	434 (93.9)	
Others	202	13 (6.4)	189 (93.6)	
**Highest Level of Education**				45.29/<0.001
Illiterate	61	12 (19.7)	49 (80.3)	
Non formal	29	24 (18.6)	105 (81.4)	
Primary	296	46 (15.5)	250 (84.5)	
Secondary	1691	144 (8.5)	1547 (91.5)	
Tertiary	1091	73 (6.7)	1018 (93.3)	
**Income**				9.71/0.01
≤RM1000	592	72 (12.2)	520 (87.8)	
RM1001 to 3000	2158	191 (8.9)	1967 (91.1)	
≥RM3001	518	36 (6.9)	482 (93.1)	
**Smoking status**				219.89/<0.001
Never smokers	2596	146 (5.6)	2450 (94.4)	
Past smokers	167	20 (12.0)	147 (88.0)	
Current smokers	505	133 (26.3)	372 (73.7)	
**Awareness on the existence of national laws prohibiting smoking in certain areas**				20.72/<0.001
Yes	2827	236 (8.3)	2591 (91.7)	
No	129	24 (18.6)	105 (81.4)	
Unsure	312	39 (12.5)	273 (87.5)	
**Knowledge of places which are smoke free according to law**				30.16/<0.001
Poor knowledge	998	133 (13.3)	865 (86.7)	
Good knowledge	2270	166 (7.3)	2104 (92.7)	
**Health effects on smokers**				122.59/<0.001
Poor knowledge	2825	103 (23.3)	340 (76.7)	
Good knowledge	443	196 (6.9)	2629 (93.1)	
**Health effects on passive smokers**				83.48/<0.001
Poor knowledge	1070	115 (18.7)	499 (81.3)	
Good knowledge	2198	184 (6.9)	2479 (75.6)	

The tolerance to smoking among the respondents was 32.7%. As shown in Table 2, there is a six fold odds of not-supporting the smoke free initiative among those tolerant to smoking in public areas. Regression analysis showed that there was a fourfold odds of not supporting the initiative among those tolerant to smoking in public areas. Tolerance towards smoking was associated with 80.3% risk of non-support in the respondents who were tolerant to smoking and a 57.2% risk in the population.

**Table 2:** Tolerance to smoking and its associated risks of non-support to making GTWHS a smoke free zone.

Variable	Prevalence	Unadjusted Odds ratio	Adjusted odds ratio	Attributable risk exposed	Attributable risk population
**Tolerance to smoking**	32.7%	6.1 (4.7;7.9)	4.0 (3.0;5.4)	80.3%	57.2%

## Discussion

Tobacco control measures have avoided an estimated 8 million premature deaths and extended the mean life span by 19 to 20 years [17]. Although Malaysia is relatively new in actively adopting tobacco control policies, the ministry of health and several Non-Governmental Organizations (NGO) have been actively and consistently disseminating information pertaining to the risks associated with smoking to the public. This could explain the high level of support for the proposed smoke free policy which is comparable with that of developed countries [18]–[21] and other developing countries like Thailand [22]. The implementation of smoke free legislation has been shown to be effective even in low and middle income countries like Malaysia. In Uruguay the exposure to second hand smoke decreased greatly in indoor public places and workplaces after the implementation of the smoke free legislation [23].

In the present study, a higher proportion of owners and residents/tenants and the Chinese compared to others were non-supportive of the smoke free zone initiative probably because most owners of the premises/businesses in the area are Chinese and they fear that this proposal may affect their businesses. The support for the initiative was higher among women which is similar to the findings of studies conducted elsewhere [10],[16],[19],[24]. This could be because the prevalence of smoking among women is much lower compared to men. The correlation between education and level of support is because of the ability to understand the information on health and the increased self-awareness leading to the decision not to support unhealthy choices [10],[21]. Similarly poor knowledge is associated with lower support of smoke free policies [10],[20],[25],[26]. Better knowledge is associated with improved understanding of the risks associated with smoking and second hand smoke. Hence it is important to create awareness of the smoke free policies which has been shown to be associated with higher intolerance to smoking [27]. Education could also be a reason for the decrease in the support among the older age groups in this study. This finding contradicts the findings of studies conducted elsewhere which showed higher support among older respondents [10],[16],[18]–[20],[24]. In a relatively young country like Malaysia the younger age groups are more aware of the dangers of smoking and second hand smoke compared to the elderly whose level of education is lower and the knowledge on the adverse effect of smoking on health is limited [25]. The support from adolescents is important because they influence each other's habits though peer influence and social norms [27]. In general, support is high among those who never smoked and lowest among current smokers [18],[21],[26]–[28]. Although smokers and non-smokers generally believe that there is a harmful effect in passive smoking and both groups support tobacco control measures, non-smokers are usually more in favour of smoking bans than smokers [29]. Furthermore smokers whose social environments condone smoking are even less likely to support tobacco control policies [10].

The authors used tolerance to smoking as an indicator for attitudes towards smoking. Gilpin [16] indicated that a population's belief where smoking should not be allowed can be considered as an indicator of its attitude towards smoking. This study found about one in every third respondents was tolerant towards cigarette smoke and the attributable risk of tolerance to non-support was high. The tolerance to tobacco smoke is also high among European Union (EU) citizens where only about half of its citizens (57%) are bothered by the exposure to second hand smoke while 30% are rarely and 27% never bothered and most were concerned about the smell rather than the health consequences [19]. People living in countries with weak smoke free policies are more likely to be exposed to second hand smoke compared with people living in countries with comprehensive smoke free policies [30]. In Thailand where the tobacco control policies are better compared to Malaysia, Thai smokers are more likely to be concerned about harm to others than Malaysian smokers are and they are also more likely to support smoke free policies [22]. In Malaysia, health education and promotion has always mainly targeted smokers in an attempt to get them to quit smoking by primarily educating them on the hazards of smoking. Very little health promotional effort is made to educate the public concerning the harmful effects of second hand smoke. This has resulted in a lower proportion of people believing that breathing other peoples smoke causes serious illness in non-smokers [4]. One of the strategies to change the attitudes towards smoking is by adopting comprehensive smoke free policies which make smoking socially unacceptable. Strong smoke free policies are associated with favourable attitudes [31]. Even with minimal initial support, the adoption of smoke free policy has been shown to increase support over time and the resistance towards such policies diminish once implemented [24],[31]–[33]. Smoke free policies which are markers for denormalization and social unacceptability of smoking [25],[34] help protect non-smokers from exposure to second hand smoke and reduce tobacco use. Such policies also encourage cessation by providing a supportive environment to quit smoking and these policies reduce tobacco initiation among young people because of the lower visibility of role models, fewer opportunities and diminished social acceptability and social advantages for smoking. These policies eventually result in improved health [2],[12],[16],[29], [35]–[44]. In the US, smoking was acceptable and considered normal prior to the 1964 surgeon general's report on the health effects of smoking. This changed after the report was published, non-smokers rights movement emerged and public policies were introduced to change the attitudes of the public to denormalize smoking by making it socially unacceptable to smoke everywhere. This resulted in reduced smoking rates and the protection of more than half of Americans from second hand smoke [2],[11],[13],[36],[45]. In California the tobacco programme aimed at changing social norms related to smoking by educating the public regarding the dangers of second hand smoke and the setting up of smoke free venues has been shown to be effective in changing population attitudes. Such was its success that smokers have been shown to be more considerate about not smoking in the presence of non-smokers. Furthermore because positive attitudes to smoke free public places stimulate adoption of smoke free homes,[46] more than half of all California smokers' homes were reported smoke free [47]. In most developed countries, as a result of the denormalisation and unacceptance of smoking in public areas, smoking habits now carry a connotation of being a filthy habit [48] and is associated with unemployment, low socioeconomic status and low education [49]. Smokers are ostracised and are obliged to smoke in unpleasant surroundings and in extreme weather conditions and are not eligible for insurance premium reductions as non-smokers [48]. These have helped reduce the prevalence of smoking and reduce the morbidity and mortality related to smoking [50].

## Conclusions

Malaysian health promotion and education on tobacco control has mainly targeted smokers. This must change; education concerning the risks of second hand smoke should also be targeted towards non-smokers. The education provided can increase the knowledge on the adverse effects of tobacco smoke which will help reduce social acceptability of smoking. This will result in the acceptance of policies restricting second hand smoke and the reduction in smoking rates [35]–[37],[51]–[54] leading to reduced adverse health consequence [2],[55],[56]. Educating the public on the dangers of second hand smoke using mass media campaigns have been shown to be effective even on people with low levels of education [16],[53]. This would be an effective tool especially among the elderly Malaysians who have lower levels of education compared with the younger population. The information given can empower non-smokers to speak out against smoking habits [34] and win the support of smokers who will better understand the risks associated with second hand smoke which can trigger acceptance of society wide anti-smoking policies [9]. Mass media campaigns aimed at restructuring perception rather than sending pure anti-use messages [57] can also be used to assure hospitality industries that there is no negative economic impact on their businesses and that sales do not decline as a result of smoke free laws [44],[58].

### Limitations

There are several limitations to the study. The main one concerns sampling. Due to the dynamic population in this study the investigators were unable to do probability sampling. However every effort was made to collect data from different levels of the sample units which included the owners or senior management, employees, clients and the general public.

### What This Paper Adds

Denormalization of smoking has been shown to be an effective method of tobacco consumption control in most developed countries. In a developing country like Malaysia the focus of health education and promotion has been towards smokers in an effort to get them to quit smoking. Although this has been shown to be successful to some extent, the high tolerance to smoking is a cause of concern. There is a need to change the tobacco control strategies by focussing the health educational efforts towards non-smokers. The denormalization of smoking will not only help reduce uptake but also increase quit rates.

## Supporting Information

Appendix S1 Scale used to assess the tolerance towards smoking.
(DOCX)
