# Supplementary material for: The Support for Smoke Free Policy and How It Is Influenced by Tolerance to Smoking – Experience of a Developing Country
Source: PLoS One. 2014 Oct 22;9(10):e109429. doi: 10.1371/journal.pone.0109429 (PMC4206272; doi:10.1371/journal.pone.0109429)
Supplement: Appendix S1 — Scale used to assess the tolerance towards smoking. (DOCX) [file pone.0109429.s001.docx]

**APPENDIX**

**Scale used to assess the tolerance towards smoking**

For each of the following places, please tell me would you tolerate smoking in this area:

| *Tick ☑ in the appropriate box* | **YES** | **NO** |
| --- | --- | --- |
| 1. Educational facilities |  |  |
| 1. Health facilities |  |  |
| 1. Non air-conditioned eating places |  |  |
| 1. Air-conditioned eating places |  |  |
| 1. Government offices |  |  |
| 1. Private air-conditioned offices |  |  |
| 1. Private non air-conditioned offices |  |  |
| 1. Hotel & accommodations |  |  |
| 1. Public vehicles (e.g. bus, ferry, taxi) |  |  |
| 1. Public transports terminals |  |  |
| 1. Places of worship/gathering for religious purposes |  |  |
| 1. Shopping complexes |  |  |
| 1. Public parks |  |  |
| 1. Stadiums, sports complexes and fitness centres |  |  |
| 1. Area use for any assembly/gathering in a building except private/ residential |  |  |
| 1. Open Market / bazaar |  |  |
